# Supplementary material for: The highly diverse plasmid population found in Escherichia coli colonizing travellers to Laos and its role in antimicrobial resistance gene carriage
Source: Microb Genom. 2023 May 12;9(5):mgen001000. doi: 10.1099/mgen.0.001000 (PMC10272864; doi:10.1099/mgen.0.001000)
Supplement: Supplementary material 1 [file mgen-9-1000-s001.pdf]

Supplementary Data

Plasmid type	Name	Genbank Accession number
FII-2	p39R861-3	MK092064.1
IncX1	pSRC22-2	MN256104
IncQ	RSF1010	M28829
FII-18	pCERC3	KR827684
IncY	P1	AF234172
ColRNAI	pCERC7	KX356458

Table S2 – Reference plasmids used to annotate key genes on plasmid types identified in this dataset

Section number	Section looked for in Illumina dataset
ColE1-like plasmid (pLAO84) MITESen1 to backbone section 1	TTAAATAATGCGCTTAACGTACAAAAAATTCCGATCTCCA AACTGACCCCTTCTTCGCCTCTTATGTTTCTCGAATGAAT CGATGGGGACAGGAAATGTT
ColE1-like plasmid (pLAO84) MITESen1 to backbone section 2	ATTTATTTTTCATGAAGTTGCGATAAAAATCGCAGCTGCG TTAGGTGTATGGGGTCAGTTTGGATATAGAGAATTATTGT ACGGTAAGCCTGTTTTTTGA
IncQ1 Section from pLAO60	CCGTTAACTGTCACGCCCCCCCGTTAACTGTCACGAACC CCCCGTTAACTGTCACGCCCCCCGTTAACTGTCACGAAC CCCCCGTTAACTGTCACGCCCCCC

Table S3 – DNA sections searched for in the Illumina dataset (Kantele et al., (7)) to look for circulating ColE1-like plasmid (pLAO84) and IncQ1 plasmid (pLAO60)

A)

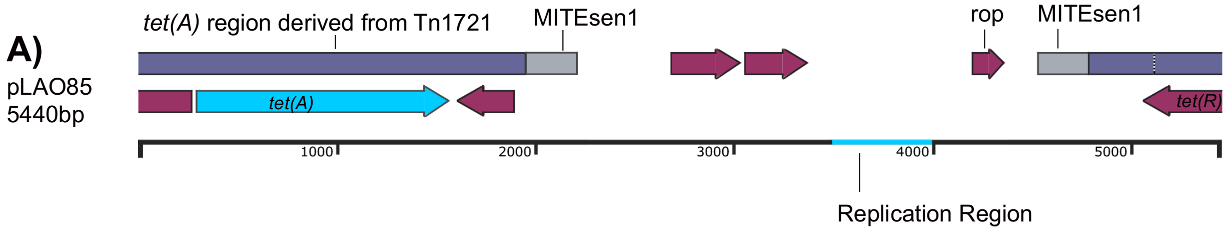

B)

**MITSen1 and MITSen2 Blast contig hit**	**Findings**
LA239	Original Known ColRNAI with tetracycline genes
LA193	'lasmid found complete on one contig
LA191	Plasmid found across two contigs
LA189	Original Known ColRNAI with tetracycline genes
LA169	Plasmid found across two contigs
LA166	Plasmid found across three contigs
LA139	Plasmid found across two contigs
LA132	Plasmid found across two contigs
LA092	Plasmid found across three contigs

Figure S1 A) Annotated ColE1-like plasmid (pLAO84) harbouring tetracycline resistance with Tn*1721* region (purple) and MITESen1(grey). Other prokka annotated genes shown in maroon. **B) Isolates in Illumina Laos Dataset (6) that contain this same ColE1-like plasmid**

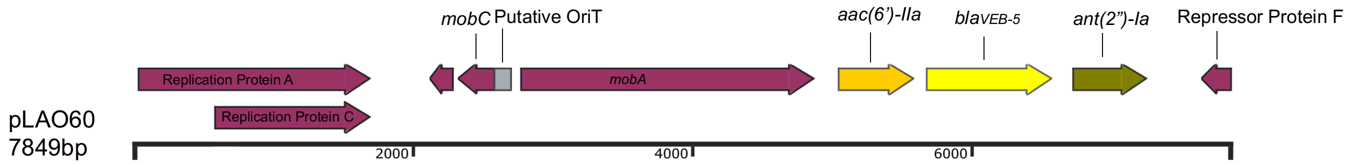

Figure S2 – Annotated IncQ1 plasmid map from pLAO60 indicating the location of bla$_{\text{VEB}}$ All maroon genes are prokka annotated genes. All other brightly coloured genes are antibiotic resistance genes.

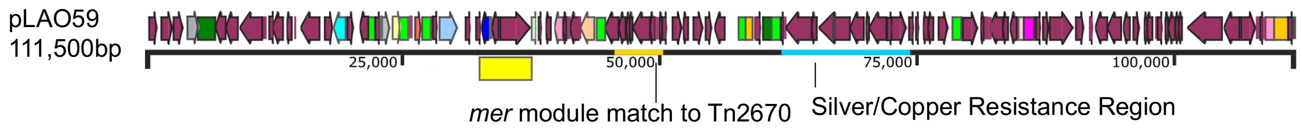

Figure S3 - Annotated IncY plasmid map (pLAO59) identifying metal resistance regions and P1-like region. All maroon genes are prokka annotated genes. All other brightly coloured genes are antibiotic resistance genes. Transposable elements (e.g. transposons, IS elements) are displayed as brightly coloured boxes. Metal resistance regions marked for copper and silver resistance(blue) and mercury resistance (orange).

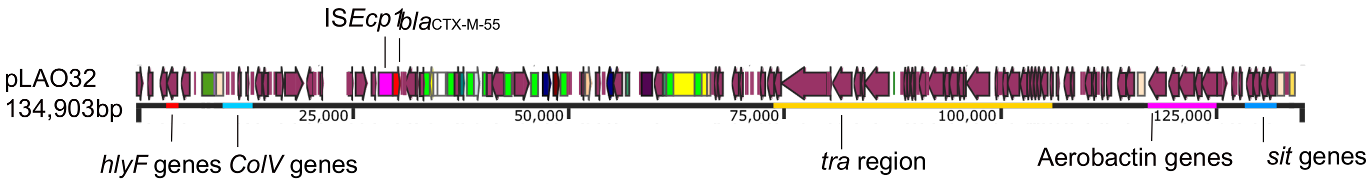

Figure S4 – Annotated FII-18 plasmid map (pLAO32) showing virulence genes and *bla*$_{\text{CTX-M}}$. All maroon genes are prokka annotated genes. All other brightly coloured genes are antibiotic resistance genes Transposable elements (e.g. transposons, IS elements) are displayed as brightly coloured boxes. Notable IS elements are IS*26* (bright green), IS*Ecp*1 (bright pink). Transfer (*tra*) region is highlighted with the yellow outline.

Plasmid	Query cover (%)	Percentage identity (%)
pLAO82	100	100
pLAO37	100	100
pLAO78	100	99.98
pLAO86	47	92.97
pLAO55	47	92.70
pLAO11	47	92.70
pLAO71	36	99.95
pLAO61	36	99.95
pLAO10	36	99.95
pLAO93	39	97.24

Table S7 – Novel transposon Tn *7514* in other pLAO plasmids that contain complete or partial Tn*7514*. This table shows query cover and percentage identity of Tn7514 found in these pLAO plasmids. This novel transposon has been registered with the Transposon Registry and was allocated the name Tn*7514*.
